# Effects of High Pressure Modification on Conformation and Digestibility Properties of Oyster Protein

**DOI:** 10.3390/molecules24183273

**Published:** 2019-09-09

**Authors:** Runfang Wang, Suisui Jiang, Yujin Li, Yunsheng Xu, Tietao Zhang, Fan Zhang, Xue Feng, Yuanhui Zhao, Mingyong Zeng

**Affiliations:** 1 College of Food Science and Engineering, Ocean University of China, Qingdao 266003, China (R.W.) (S.J.) (Y.L.) (F.Z.) (X.F.); 2 College of Food Science and Engineering, Hainan Tropical Ocean University, Sanya 572022, China (Y.X.) (T.Z.)

**Keywords:** oyster protein, high pressure, conformation, microstructure, zeta potential, digestibility

## Abstract

To expand the utilization of oyster protein (OP), the effects of high pressure (100 to 500 MPa) on chemical forces, structure, microstructure, and digestibility properties were investigated. High pressure (HP) treatment enhanced the electrostatic repulsion (from −13.3_Control_ to −27.8_HP200_ mV) between protein molecules and avoided or retarded the formation of protein aggregates. In addition, the HP treated samples showed uniform distribution and small particle size. The changes in electrostatic interaction and particle size contributed to the improvement of solubility (from 10.53%_Control_ to 19.92%_HP500_ at pH 7). The stretching and unfolding of protein were modified by HP treatment, and some internal hydrophobic groups and -SH groups were exposed. HP treatment modified the secondary structure of OP. The treated samples contained less α-helix and β-sheet structures, whereas the proportions of β-sheet and random coil structures were increased. The treated samples have high digestibility in the stomach (from 26.3%_Control_ to 39.5%_HP500_) and in the total digestive process (from 62.1%_Control_ to 83.7%_HP500_). In addition, the total digestive production showed higher percentages of small peptides (<1 kDa) after HP treatment. The protein solubility and digestibility were increased after HP treatment, and high solubility and high digestibility might increase the chance that OP become a kind of protein supplement.

## 1. Introduction

Oyster is an abundant resource from ocean [1], containing a low content of fat and high content of vitamins and minerals [2]. It is noteworthy that it contains more than half protein by dry weight [3], and the content of essential amino acid is higher than the value recommended by FAO/WHO. There are some shortages in conventional water extract method for oyster protein extraction, such as the low extraction rate and complex separation process [4,5]. Some authors have reported the protein extraction technology of isoelectric solubilization/precipitation (ISP); this method could simplify the extraction process and improve the extraction rate with high purity [6]. This method makes protein denaturation, due to extreme pH shifts during ISP, containing poor solubility, emulsifying property, and foaming ability. 

High-pressure (HP) treatment has seen significant developments, and it is used in the food system to modify the properties of proteins [7,8]. Puppo et al. [9] reported that the solubility of soybean bran increased from approximately 58% to 78% when pressure above 200 MPa was applied. HP treatment could affect the chemical forces between protein molecules; it can increase the surface hydrophobic activity [10], improve the electrostatic interactions between protein molecules [11], and change the content of -SH [12]. In addition, the modification of protein tertiary and quaternary structures could be induced by HP treatment, which further affected the functional properties [13]. Some authors have found that the functional properties of water retention capability and oil retention capacity are improved by the changing of physical structure [14]. HP treatment could change the physical properties of rapeseed protein, further leading to changes in its gelation properties [10].

Although HP treatment has been used to improve the functional properties of proteins, there is little information on its application to animal protein, especially aquatic product protein. We assume that HP treatment could improve the solubility and some other physical properties of the oyster protein, and that it could further improve the functional properties and protein utilization. The purpose of this work was to investigate the effects of HP treatment on the chemical forces, structure, and microstructure of oyster protein (OP), and its influence on solubility and digestibility. Based on the findings, we attempt to find out a better way to expand the utilization of oyster protein.

## 2. Results and Discussion

### 2.1. Zeta Potential, Particle Size Distribution, and Solubility

**Figure 1:**
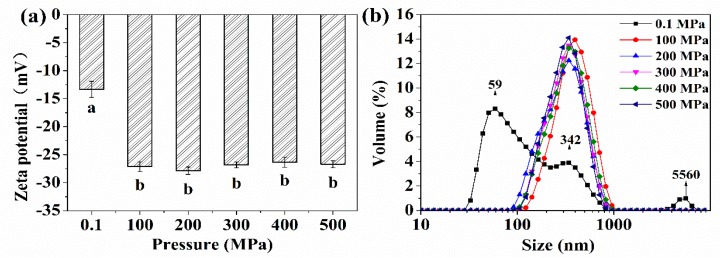
The zeta potential (**a**) and size distribution (**b**) of control and HP treated oyster proteins. Different lowercase letters indicate significant difference between groups (*p* < 0.05).

As shown in Figure 1a, the electrostatic interactions between protein molecules was quantified by using the zeta potentials of the OP solutions at pH 7. The lowest zeta potential (−13.33 mV) was found for untreated OP, which indicates that the electrostatic interactions between protein molecules was weak. It is clear that the zeta potential increased twice after HP treatment, which could be induced by more exposure of ionizable acids on the protein surface after HP treatment [7]. The observation was in agreement with Chen et al. [11]; the author found that the zeta potential of protein increased after HP treatment. In addition, the dissociation of an amino acid group could also result in an increase in protein charge [15].

The particle size distribution of protein is shown in Figure 1b. The untreated sample showed a polymodal distribution with peaks at 59 nm (68.0%), 342 nm (25.4%), and 5560 nm (2.8%), respectively. The HP treatment narrowed the particle size distribution, and all treated samples showed a single peak; when the pressure was 100 MPa, the peak appeared at 396 nm, and the peak was at 342 nm when the pressure was above 200 MPa. After HP treatment, protein molecules larger than 1000 nm were hardly observed. HP treatment could make the complex macromolecular structure dissociate and fragment into small particles, which would markedly reduce the large aggregation of protein [9,16]. The smallest peak of the untreated sample was 59 nm, but this peak disappeared, and only the second smallest peak (342 nm) could be observed after treatment. The increase protein particle size of the second smallest peak was probably due to the aggregation of the smaller molecules, which is attributed to the intermolecular disulfide bridges and hydrophobic interactions [13]. Conclusively, the result showed that the OP has undergone disruption and breakage under HP treatment. 

The solubility changes in the OP samples under various pH levels (changing from 3 to 10) are shown in Table 1. For all samples at the same pressure level, the solubility of OP decreased from pH 3 to 4 and increased from pH 4 to 10. These results are in agreement with those of Yu et al. [15], the isoelectric point (pH 4.5–5.1) may be the important mechanism for the lowest values around pH 4 to 6.

The solubility of OP differed between the control and HP-treated samples when compared at the same pH level. When the pH ranged from 2 to 9, the solubility of the HP-treated samples increased significantly. For example, at pH 7, the solubility was 10.53%_Control_, 12.18%_HP100_, 15.19%_HP200_, 18.85%_HP300_, 20.83%_HP400_, and 19.92%_HP500_, respectively. On the other hand, there was no significant difference between the solubility when the pressure was above 300 MPa. According to the reports, the protein conformation, particle size, exposed charged amino and carboxyl groups were important reasons to explain the increased solubility of proteins under HP treatment [8,17,18]. It is clear that the net negative charge increased after HP treatment, as seen in Figure 1a. The enhanced electrostatic repulsion between protein molecules may avoid and delay protein aggregation and improve the protein solubility [11]. HP treatment can lead some complex macromolecular proteins to dissociate into small particles [16]. As shown in Figure 1b, the particle size of the HP-treated samples was more uniform than that of the control sample.

### 2.2. The -SH Groups and Surface Hydrophobicity

**Figure 2:**
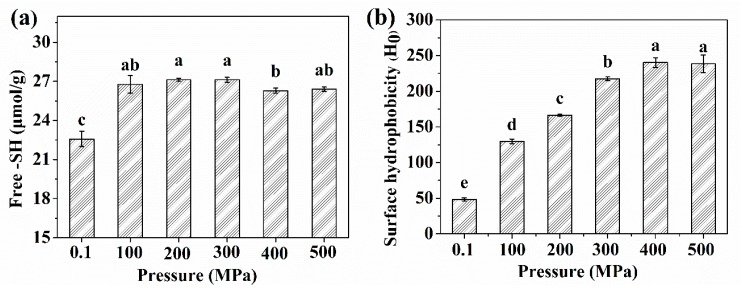
The content of free -SH (**a**) and surface hydrophobicity (**b**) of control and HP treated oyster proteins. Different lowercase letters indicate significant difference between groups (*p* < 0.05).

The -SH groups belong to a kind of covalent bonds and play a role in maintaining the protein tertiary structure [19]. Changes in the free -SH content of protein are shown in Figure 2a. The free -SH content increased significantly after HP treatment. The concentration of free -SH content in the control and HP300 were 22.58 and 27.12 μmol/g. These observations are in agreement with those of Wang et al. [8], the author found that HP treatment (200 to 600 MPa) results in increased free -SH content.

Some authors have speculated that the increase in free -SH content maybe induced by the breaking of disulfide bonds [20], whereas others have reported that the energy was only 8.37 kJ/mol provided by 10,000 MPa, while the required energy was 213.1 kJ/mol to disrupt the covalent bonds, such as disulfide bonds [13]. Protein stretching and unfolding contributed to the exposure of inter -SH groups under HP treatment, and the free -SH content was increased. In fact, in native OP, some -SH groups were covered and could not be attacked by Ellman's reagents because of protein folding. After HP treatment, the inter -SH groups would be exposed to the external environment [12].

The hydrophobic interactions between protein molecules are important in maintaining protein stability, conformation, and functional properties [21]. As shown in Figure 2b, the H_o_ of samples at pH 7.0 were evaluated using ANS as the fluorescence probe. Compared to untreated OP, HP treatment resulted in significant increases of H_o_ from 100 to 500 MPa. When the pH level was above 300 MPa, the highest H_o_ (240.39) was observed. Zhang et al. [13] obtained similar experimental results for the HP treatment of myofibrillar protein and reported that the relative fluorescence intensity of ANS increased with increasing pressure.

The increased H_o_ could be induced by the dissociation of protein. As seen in Figure 6, it is clear that the morphological structure of the protein was changed. The unfolding and extension of the treated OP peptide chains could expose many hydrophobic groups or non-polar active binding sites; ANS molecules were closer to the buried hydrophobic core between protein molecules [10,22]. The hydrophobic groups were discovered in the interior of the protein, and HP treatment caused more hydrophobic groups to be exposed to the surface of protein [9,23]. Moreover, the protein solution interactions, intra-protein molecules, and protein–protein interactions could also suggest the increase of H_o_. 

### 2.3. UV–Vis Spectrum and Intrinsic Fluorescence Spectroscopy

**Figure 3:**
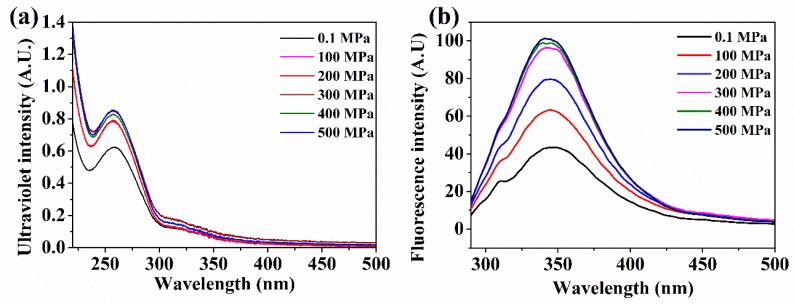
The UV scanning spectrum (**a**) and intrinsic fluorescence spectra (**b**) of control and HP treated oyster proteins.

The UV–Vis spectra of the OP samples are shown in Figure 3a. The peaks were located in the near–UV region (from 240 to 300 nm). The maximum ultraviolet absorption was 288 nm, and the intensity of the absorption peak increased significantly after HP treatment. For example, the intensity of the peak was 0.624; it became 0.854 when the pressure was 500 MPa. The peaks of UV absorption is usually ascribed to the aromatic amino acids residues (Trp, Tyr, and Phe) and the S–S bonds that constitute chromophores with a strong absorption peak [24]. In addition, the emission peak of the fluorescence spectra is also mainly attributed to aromatic residues, particularly Trp. In addition, the maximum emission peak is determined by the polar environment [8,25].

As seen in Figure 3b, the increasing emission peak of the fluorescence spectra could be induced by the protein unfolding and the exposure of more chromophores to the external environment. The control sample showed the maximum emission peak at the wavelength of 348 nm. After HP treatment, the intensity of the emission peak increased significantly, and the peaks were 345_HP100_, 345_HP200_, 343_HP300_, 340_HP400_, and 341_HP500_ nm, respectively. A blue shift was clearly observed after HP treatment. These results are in agreement with those of Yin et al. [26], the author found a similar increase in fluorescence emission intensity with increasing pressure. It is recognized that the fluorescence intensity of Trp is quenched by a polar solvent, and the changing peaks indicate the exposure of more hydrophobic groups from the interior of the molecule [14]. The results showed that conformational changes in the tertiary and quaternary structure levels could be more sensitive and more easily changed at high pressures, which causes the exposure of hydrophobic groups.

### 2.4. Fourier Transform Infrared Spectroscopy (FTIR)

**Figure 4:**
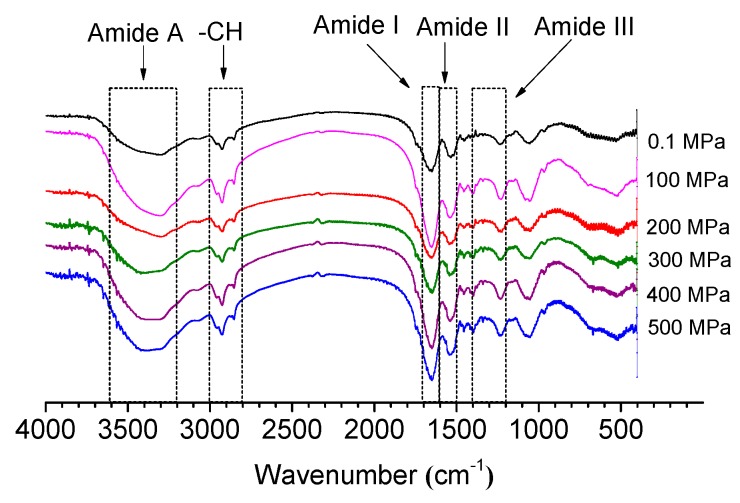
The FTIR spectrum of control and HP treated oyster proteins.

To evaluate the effect of HP treatment on the protein secondary structure, the FTIR spectral analysis was carried out to determine the changes in protein at various pressure levels. The observed FTIR spectra are shown in Figure 4. Eleven peaks were observed in the spectra of non-treated OP, namely, at 961, 1056, 1172, 1224, 1369, 1454, 1517, 1648, 2854, 2926, and 3322 cm^−1^. The FTIR spectra reflects the Amide A strength information in the range from 3200 to 3600 cm^−1^ and the –CH bond stretching vibrations ranging from 2800 to 3000 cm^−1^. In addition, the amide I band (1600 to 1700 cm^−1^) is due to the C=O stretching vibration of the peptide bond, and the amide II band (1500 to 1600 cm^−1^) is mainly ascribed to C–N stretching and N–H bending from amide groups [14,27,28].

#### 2.4.1. Influence of HP Treatment on Amide I Bands and Amide II Bands

According to some reports, both amide I and II are both sensitive to changes in the protein secondary structure, which could reflect the vibrational bands of the protein backbone to some extent. The contribution of amide II for quantification of the secondary structure of proteins is very large [13,29]. According to a previous report, the bands at 1617 to 1623 cm^−1^ and 1691 to 1698 cm^−1^ can be ascribed to β-sheets, and the bands between 1667 and 1685 cm^−1^ are due to β-turns. However, the bands at 1636 to 1643 cm^−1^ and 1647 to 1658 cm^−1^ are attributed to random coils and α-helix, respectively [30]. In Table 2, the contents of α-helices, β-sheets, β-turns, and random coils are summarized. When the pressure was 500 MPa, the sample showed lower α-helix content (from 28.27% to 25.66%) and β-sheet content (from 15.68% to 11.90%). On the other hand, the β-turn and random coil contents increased from 31.16% to 33.75% and from 24.89% to 28.68% (*p* < 0.05), respectively. The α-helices are mainly maintained by hydrogen bonds from carbonyl oxygen (–CO) and amino hydrogen (–NH), which are mainly hidden inside protein molecules; however, random coils are derived from the unfolding of protein tertiary and quaternary structure and involved with protein flexibility [11]. These results showed that the secondary structure was modified by HP treatment. 

#### 2.4.2. Influence on Amide A and –CH under HP Treatment

All samples had weak peaks in the range of amide A. This phenomenon could be ascribed to intermolecular H–bonded N–H and O–H stretching vibration [31]. When the wavelength was changed from 2800 to 3000 cm^−1^ (–CH), there were two strong peaks in the untreated sample, which could be ascribed to the absorption of sugar units. This observation is in agreement with that of Liu et al. [32]; the authors found that there were two typical absorption peaks (2926 and 2962 cm^−1^) in the glycoprotein of oyster juice. You et al. also found that there was a C–H band at around 2963 cm^−1^ in the protein of pearl oysters [33]. We speculate that the sample contains glycoprotein according to the stretching vibrations of the –CH bond and the pyranose ring. It should be highlighted that HP treatment frequently leads to shifts in the FTIR spectra when the wavenumber changed from 2800 to 3000. The absorption peak of –CH stretching vibration was 2854 cm^−1^ in non-treated protein; it shifted to 2853 cm^−1^ after 400 and 500 MPa treatment. The absorption peak changed from 2926 to 2925 cm^−1^ when the pressure was 400 MPa. We speculate that the HP treatment may have an influence on the glycoprotein present in the sample.

### 2.5. Sodium Dodecyl Sulfate Polyacrylamide Gel Electrophoresis (SDS-PAGE) 

**Figure 5:**
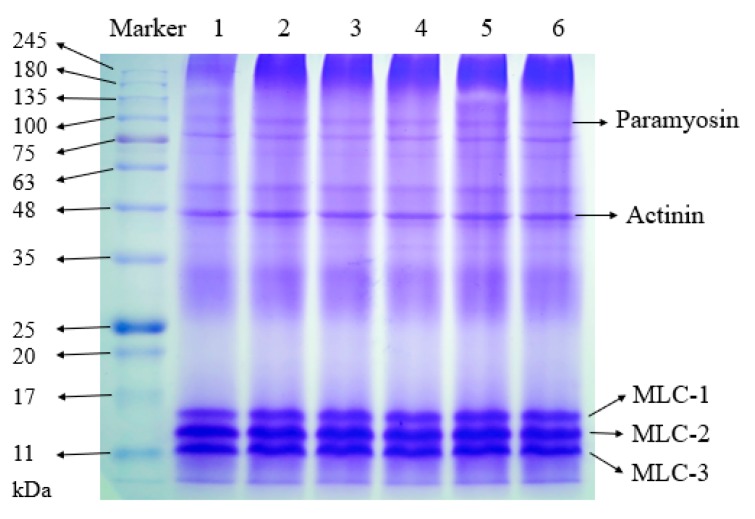
SDS-PAGE of control and HP treated oyster proteins under reducing conditions. Lanes 1–6 represent 0.1, 100, 200, 300, 400, and 500 MPa, respectively.

The molecular weight distribution of the OP samples is shown in Figure 5. The samples showed similar characteristics under the reducing conditions. The typical protein profiles were observed in OP: paramyosin (98 kDa), actin (43 kDa), and myosin light chain (MLC, 11–20 kDa) [11,34,35]. The molecules over 200 kDa aggregated, the large molecules could be blocked and could not pass but accumulated in the gel pores. All samples showed deformed bands at the beginning of the funing gels. The 55 kDa band was mainly characterized of the oyster, which was agreement with the findings of a previous report [36]. 

### 2.6. Microstructure Analysis

**Figure 6:**
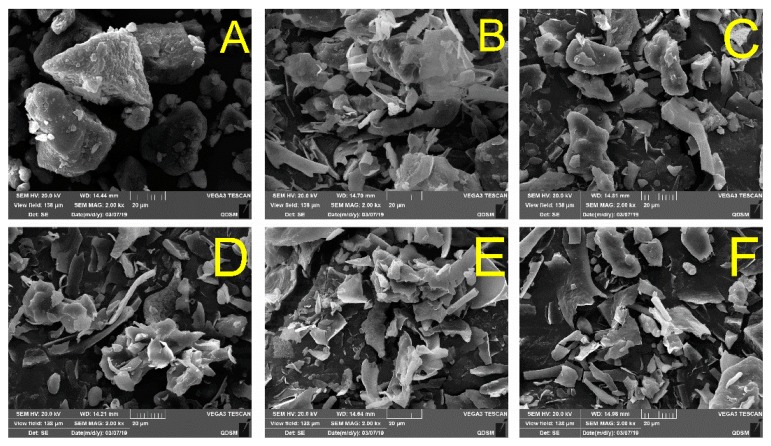
SEM images of (**A**) control and HP-treated oyster proteins by (**B**) 100 MPa, (**C**) 200 MPa, (**D**) 300 MPa, (**E**) 400 MPa, and (**F**) 500 MPa, respectively.

The microstructure of the OP samples was observed to study the appearance change, as shown in Figure 6. Before HP treatment, the microstructure of OP showed massive structures with a rough appearance and a distinct tendency to aggregate (Figure 6A). Our observation is similar to the findings of previous reports, the authors found that the chicken myofibrillar protein showed large particle size with irregular geometry before HP treatment [11]. Some large particle protein seemed to be destroyed when the pressures were 100 MPa and 200 MPa, while some protein with block structure were still retained (Figure 6B). The appearance of the samples became more irregular and extensively disrupted, and the massive structures became flake-like lamella when the pressures were above 300 MPa (Figure 6E,F). Some large blocky protein eventually disintegrated into smaller particles when the pressure was 500 MPa. Our results agree with previous reports, authors reported that the original flake-like structure of myofibrillar protein and pectin were broken into smaller chips after HP treatment [11,37,38]. The observation indicated that the strong physical force induced by HP treatment could cause severe appearance changes in OP.

### 2.7. In Vitro Digestibility and MW Distribution of Peptides

**Figure 7:**
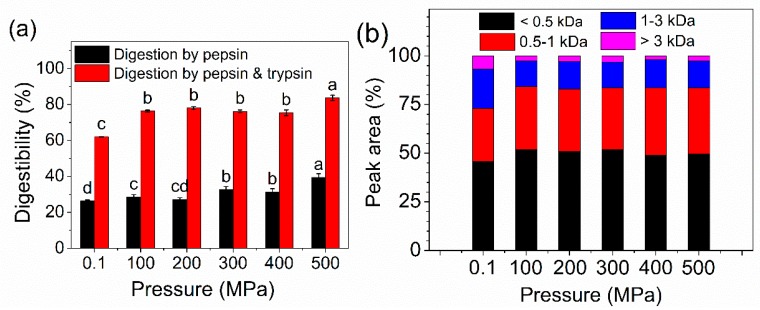
The digestibility (**a**) and the MW distribution of total digestive production (**b**) of control and HP-treated oyster proteins. Different lowercase letters indicate significant difference between groups (*p* < 0.05).

The ability of pepsin and the combination of pepsin and trypsin to digest OP increased after HP treatment, as shown in Figure 7a. All HP–treated samples showed higher digestibility compared to the control sample. The digestibility by pepsin increased gradually with increasing pressure. On the other hand, there was no significant difference between the four HP–treated samples (100 to 400 MPa). Overall, the HP–treated samples were easier to be digested compared to untreated samples. It was reported that HP–treated β-lactoglobulin was easier to digest than untreated samples because the digestion sites of pepsin are aromatic amino acids and hydrophobic amino acids [39,40]. As previously discussed, more hydrophobic groups are exposed to the external from the interior of the molecule, indicating that the hydrolysis activity of pepsin was enhanced after pressurization [41]. The increased protein solubility could afford more contact area between protein and solution, which could contribute to the improvement of digestibility. In addition, trypsin is sensitive to the structure of protein; hence, the susceptibility of trypsin hydrolysis was considered as an index of structural integrity for some proteins [26]. HP treatment destroyed the protein structure accompanied by unfolding of OP and exposure of some inter groups, which may improve the hydrolysis activity of trypsin. 

The MW distribution profiles of total digestive production was presented in Figure 7b. MW distribution ranges include <0.5 kDa, 0.5–1 kDa, 1–3 kDa, and >3 kDa. All digestive production from control and HP-treated samples were mainly composed of small peptides (<1 kDa). It should be highlighted that HP-treated samples could release higher percentages of low MW fractions (<1 kDa) compared to the control sample, and the percentage was 72.87%_Control_, 84.28%_HP100_, 83.01%_HP200_, 83.57%_HP300_, 83.71%_HP400_, 83.47%_HP500_, respectively. The proteases or peptidases could digest protein (long-chain) to small peptides, then the variations of molecular weights are considered to be affected by different hydrolysis processes [42,43]. As seen in Figure 2b and discussed in Section 2.2, the hydrophobic groups were exposed after HP treatment. According to a previous report, the exposed hydrophobic regions could be degraded by pepsin and soluble fragments were formed [44,45]. Therefore, we speculate that the degree of hydrolysis increased after HP treatment and more soluble fragments were formed, the increased soluble peptide was an important reason to explain the increased digestibility. On the other hand, higher percentages of low small peptides (<1 kDa) could also be induced by high degree of hydrolysis and increased the soluble fragments. The observation was in agreement with Fu Y et al [46], they reported that small peptides (<1 kDa) and free amino acids released from beef were increasing with higher degree of hydrolysis.

## 3. Materials and Methods 

### 3.1. Materials

Fresh oysters (*Crassostrea gigas*) were obtained from the local aquatic product market (Qingdao, China). Bovine serum albumin (BSA) and the BCA Protein Assay Kit were obtained from Beijing Solarbio Science & Technology Co. Ltd. (Beijing, China), and 1- anilino-8-naphthalene-sulfonate (ANS) was obtained from Sigma-Aldrich Co. Ltd. (Shanghai, China). All other chemicals were analytical grade or better.

### 3.2. Preparation of OP

OP was extracted from fresh oysters by alkaline solution, followed by isoelectric precipitation, according to the procedure reported by Zheng et al. [6] with some modifications. Fresh oyster meat of 4000 ± 106 g in weight was used in each experiment. All results were repeated for three times. Before extraction, the oyster meat was homogenized, and then dispersed in distilled water (1:3, *w*/*v*). The pH of the mixture was adjusted to 12.5 using 0.1 M NaOH, stirred for 3 h at 4 °C, and then centrifuged at 6000 *g* for 25 min at 4 °C. Next, the pH of the supernatant was adjusted to 4.8 using 0.1 M HCl, and then centrifuged at 6000 *g* for 25 min to precipitate the protein. The precipitate was dissolved in distilled water. Its pH was adjusted to 7, and then freeze-dried (Scientz-10ND, Ningbo Scientz Biotechnology Co., Ltd., Ningbo, China) for 48 h. The freeze-dried sample was stored until further use. The protein content was 72% (*w*/*w*, dry weight), as measured using a nitrogen analyzer. 

### 3.3. High-Pressure Treatment

HP treatment was carried out using high hydrostatic pressure equipment (HPP600MPa/30L, Jiujiu Technology Development Co., Ltd, Baotou, China). Freeze-dried oyster protein of 60 g in weight was used for one trial. Before HP treatment, samples of oyster protein dispersions (2%, *w*/*v*) were stirred for 20 min at room temperature, and the solution was vacuum packed in polyethylene bags. The packed solution was treated at various pressures (100 to 500 MPa) for 10 min (expressed as HP100, HP200, HP300, HP400, and HP500). After pressure treatment, the treated samples were collected and freeze-dried until further use. The OP sample without pressurization was treated as a control (0.1 MPa).

### 3.4. Measurement of Protein Solubility 

The solubility of the OP samples was measured using the method described in a previous report by Yang et al. [25] with some modifications. OP samples were dispersed in deionized water (1%, *w*/*v*) using a vibrator for 2 min. Then the pH was individually adjusted within the range of 3 to 10 with 0.1 M HCl or NaOH and stirred for 1 h at 25 °C. The OP solution was centrifuged at 5000 *g* for 15 min. The supernatants were collected and the BCA Protein Assay Kit was used to determine the protein content (Powerwave XS; BioTek Instruments, Inc., Winooski, Vt, USA), and BSA was used as the standard. Solubility was calculated as the percentage of soluble protein in supernatant relative to total protein content in samples.

### 3.5. Measurement of Particle Size Distribution and Zeta Potential

The particle size and zeta potential of the OP samples were determined while using the procedure reported by Chen et al. [47] with some modifications. The protein samples were diluted (0.5%, *w*/*v*) in deionized water. Then the solution was filtered with a 0.45 μm filter membrane to remove large particles. After that, the solution was injected into clear test cells to measure particle size and zeta potential, respectively. The particle size and zeta potential were determined to use a Zetasizer (Nano-zs90; Malvern Instruments Co. Ltd. Malvern, UK).

### 3.6. Measurement of Sulfhydryl Group Content

The free sulfhydryl (-SH) group contents of the OP samples were tested using the method described by Segat et al. [48] with slight modifications. The free -SH group was measured by dissolving the samples in Tris–glycine buffer (containing 0.086 M Tris, 0.09 M glycine, and 4 mM EDTA; pH 7.0). An aliquot (2.0 mL) of OP solution (10 mg/mL) was mixed with 50 mL of Ellman’s reagent (4 mg/mL DTNB in Tris–glycine buffer). The mixture was incubated for 25 min at 40 °C and then measured at the wavenumber of 412 nm by UV-Vis spectrophotometry (UV-2550; Shimadzu Crop, Tokyo, Japan), and the buffer was used as a blank. The concentration of free sulphydryl groups (μM -SH/g) was calculated by:

μM -SH/g = (73.53 × A_412_ × D)/C (1)

where A_412_ represents the absorbance at 412 nm; C is protein concentration of the sample solution (mg/mL), D represents the dilution factor, and the factor 73.53 is originated from 10^6^(1.36 × 10^4^); and 1.36 × 10^4^ represents the molar absorptivity constant.

### 3.7. Measurement of Surface Hydrophobicity

H_o_ of OP was measured by the method of Cui et al, [49] and ANS was used as the fluorescence probe. The samples were diluted (0 to 1 mg/mL) in a 0.01 M phosphate buffer (0.6 M KCl, pH 7.0). Then aliquots (20 μL) of ANS (8.0 mM in the same buffer) were added to the sample solution (4 mL). Fluorescence intensity was tested by fluorescence spectrometry (Model RF-1501, Shimadzu Corp., Tokyo, Japan). The excitation wavelength was set at 365 nm, and 480 nm for emission wavelength. The initial slope of the curve made by fluorescence intensity and protein concentration (calculated by linear regression analysis) was used as the value of H_o_.

### 3.8. UV–Vis Spectra

Protein solutions (1 mg/mL) of the OP samples were diluted in a 10 mM phosphate buffer (0.6 M KCl, pH 7.0). The UV–Vis adsorption spectra were measured by spectrophotometry (UV-2550; Shimadzu Crop, Tokyo, Japan). The spectral acquisition range was from 200 to 500 nm, and the spectra were collected at intervals of 0.2 nm.

### 3.9. Intrinsic Fluorescence Spectroscopy

The intrinsic fluorescence of the OP samples was measured by spectrophotometry (Model RF-1501, Shimadzu Corp., Tokyo, Japan) at 22 °C. Protein solutions (1 mg/mL) were prepared in a 10 mM phosphate buffer (0.6 M KCl, pH 7.0). The protein solutions were excited at 290 nm, and their emission spectra were recorded from 290 to 500 nm. A slit of 5 nm was set using both excitation and emission.

### 3.10. Fourier Transform Infrared Spectroscopy (FT-IR)

The OP samples were mixed with KBr using the ratio of 1:100 and then pressed into pellets. All spectra in the region of 4000 to 400 cm^−1^ were scanned 64 times on an FTIR spectrometer (Nicolet iS10; Thermo Scientific Corp., Madison, WI, USA).

### 3.11. SDS-PAGE

The OP samples were examined by SDS-PAGE (12% polyacrylamide) according to the method of Laemmli et al. [50] using the Mini Gel 2D (JY-ZY5, Beijing Junyi Dongfang Electrophoresis Equipment Co. Ltd., Beijing, China) electrophoresis equipment. The OP solutions (2%, *w*/*v*) were added with sample buffer to reach a final protein concentration of 2 mg/mL and heated at 100 °C for 10 min before loading into the gel. Each gel lane was loaded with 20 μL of samples or 10 μL markers. The gels were run at 80 V for approximately 40 min and then at 120 V for approximately 50 min. After electrophoresis, the gel was dyed with 0.1% Coomassie blue (R-250) in a 9:2:9 (ethanol: acetic acid: water) solution and destained in 10% acetic acid (ethanol: acetic acid: water, 1:1:8, *v:v:v*).

### 3.12. Scanning Electron Microscopy (SEM) Observations

Powdery OP samples were fixed on an SEM specimen stub using a double-sided adhesive tape, then coated with a thin layer of gold before texting. After that, the morphology of the sample was observed by SEM (JSM-5800 LV, JEOL Ltd., Tokyo, Japan) at an accelerating voltage of 20 kV.

### 3.13. In Vitro Digestibility 

The OP samples were in-vitro-digested by the procedure of Tavares et al. [51], with slight modifications. Digestion of OP was simulated using both pepsin and trypsin in vitro. The exact enzyme activity of pepsin and trypsin are 13,000 BAEE units/mg protein and 200 BAEE units/mg protein, respectively. For pepsin digestion, the protein (1 g) was dissolved in 40 mL of deionized water using a vibrator for 2 min. Then the solution was adjusted to 2.5 using 1 M HCl, and pepsin (≥400 units/mg of protein) was added. The solution was incubated at 37 °C for 2 h with continuous shaking (200 rpm/min). The pepsin was inactivated by adjusting the pH to 8. The solution was centrifuged at 5000 *g* for 15 min. For trypsin digestion, the solution described above was adjusted to 8 with 1 M NaOH, and the trypsin (1.645 units/mg protein) was added. The solution was digested under the same conditions described above and was then centrifuged at 5000 *g* for 15 min. The trypsin was inactivated by heating the reaction system in 100 °C water bath for 10 min. Then the supernatant and precipitate were separated and then freeze-dried. The weight change of insoluble protein was used to calculate the degree of digestibility. The degree of digestibility (DT) was calculated as:

DT = (1 − W_i_/W_t_) × 100% (2)

where DT represents the digestibility of protein, W_i_ is the weight of dried insoluble protein, and W_t_ is the total weight of the sample before digestion.

### 3.14. Estimation of Peptide MW Distribution

MW distribution of digestive production (supernatants) was analyzed by size exclusion chromatography (TSKgel G2000SWXL, Tosoh Corp., Tokyo, Japan) on a high performance liquid chromatography (HPLC) system (LC-AT20, Shimadzu Crop, Tokyo, Japan). Data were processed and acquired via Labsolutions software. Twenty microliters of each digestive production (2 mg/mL) were injected, eluted using 45% acetonitrile containing 0.1% trifluoroacetic acid at a flow rate of 1 mL/min, and monitored at 214 nm. The MW calibration curve was plotted using the following standards, GLY (75 Da), Glutathione (307 Da), Bacitracin (1422 Da), Insulin (5733 Da), and Cytochrome c (12,400 Da). The MW was calculated, as follows:

LogMW = −0.4374t + 6.6199(3)

MW represents MW (Da) and t means elution (min).

### 3.15. Statistical Analysis

All values in the present study are expressed as the mean ± SD of triplicate measurements. The results were tested using one-way analysis of variance with the least-significant-difference-test (SPSS 17.0 software, SPSS Inc., Chicago, IL, USA). The least significant differences (*p* < 0.05) among the treatments were accepted by Duncan’s test.

## 4. Conclusions

This study evaluated the conformation structure changes of oyster protein and its effect on the digestibility under high pressure. The HP–treated samples showed smaller particle size and higher net negative charge, HP treatment had a significant influence on improving the protein solubility. Structural changes were found in the secondary structures, in which the loss of α-helices and β-sheets with formation of β-turns and random coils was observed. The protein unfolding and extension of peptides exposed some internal groups after HP treatment, including some inter -SH groups and hydrophobic groups. Therefore, the hydrolysis processes were affected after HP treatment, and the HP treated samples showed higher digestibility with higher percentages of low MW fractions (<1 kDa). As demonstrated in this study, the application of HP treatment on oyster protein could be used to improve the protein solubility and digestibility, and more work is worthy to be done to reveal the peptide sequence of digestive production and its potential functional properties.

## Figures and Tables

**Table 1:** Protein solubility of control and HP treated oyster proteins under different pH levers.

pH	0.1 MPa	100 MPa	200 MPa	300 MPa	400 MPa	500 MPa
3	5.74 ± 0.48^c^	8.12 ± 0.22^b^	9.78 ± 0.11^a^	10.67 ± 0.99^a^	9.93 ± 0.06^a^	9.92 ± 0.48^a^
4	2.96 ± 0.34^a^	2.67 ± 0.22^a^	2.95 ± 0.23^a^	3.16 ± 0.15^a^	2.76 ± 0.19^a^	3.38 ± 0.83^a^
5	3.12 ± 0.41^e^	6.32 ± 0.34^d^	6.75 ± 0.11^cd^	9.16 ± 0.21^a^	7.44 ± 0.15^bc^	7.57 ± 0.12^b^
6	4.62 ± 0.79^e^	10.91 ± 0.24^d^	13.73 ± 0.10^c^	18.32 ± 0.25^b^	19.84 ± 0.14^a^	19.70 ± 0.1^a^
7	10.53 ± 0.96^e^	12.18 ± 0.20^d^	15.19 ± 0.10^c^	18.85 ± 0.31^b^	20.83 ± 0.27^a^	19.92 ± 0.14^ab^
8	12.57 ± 0.12^b^	14.73 ± 0.22^b^	15.60 ± 0.42^b^	19.76 ± 0.16^a^	21.62 ± 0.70^a^	20.18 ± 0.29^a^
9	17.41 ± 0.15^c^	17.98 ± 0.50^c^	18.52 ± 0.58^c^	21.75 ± 0.27^b^	24.26 ± 0.13^a^	24.18 ± 0.54^a^
10	23.22 ± 0.54^b^	21.78 ± 0.14^bc^	20.42 ± 0.29^c^	23.90 ± 0.68^b^	28.05 ± 0.97^a^	28.92 ± 0.32^a^

The different letters in the same line indicate significant differences (*p* < 0.05).

**Table 2:** The secondary structure content estimated from deconvoluted FTIR spectra of control and HP treated oyster proteins.

Sample	α-Helix	β-Sheet	β-Turn	Random Coil
0.1 MPa	28.27 ± 0.47^a^	15.68 ± 0.67^a^	31.16 ± 0.16^b^	24.89 ± 0.19^b^
100 MPa	27.15 ± 0.10^ab^	12.54 ± 0.98^b^	31.77 ± 0.29^b^	28.54 ± 0.64^a^
200 MPa	27.05 ± 0.29^b^	12.54 ± 0.23^b^	32.05 ± 0.47^a^	28.36 ± 0.67^a^
300 MPa	26.51 ± 0.36^bc^	12.14 ± 0.01^b^	33.24 ± 0.63^a^	28.11 ± 0.54^a^
400 MPa	26.35 ± 1.01^bc^	11.91 ± 0.17^b^	33.33 ± 1.26^a^	28.40 ± 0.33^a^
500 MPa	25.67 ± 0.97^c^	11.90 ± 0.46^b^	33.75 ± 0.47^a^	28.69 ± 0.73^a^

The different letters in the same column indicate significant differences (*p* < 0.05).
